# Donor-derived urologic cancers after renal transplantation: A retrospective non-randomized scientific analysis

**DOI:** 10.1371/journal.pone.0271293

**Published:** 2022-09-21

**Authors:** Vivan Hellström, Gunnar Tufveson, Angelica Loskog, Mats Bengtsson, Gunilla Enblad, Tomas Lorant

**Affiliations:** 1 Department of Surgical Sciences, Section of Transplantation Surgery, Uppsala University Hospital, Uppsala, Sweden; 2 Department of Immunology, Genetics and Pathology, Uppsala University Hospital, Uppsala, Sweden; 3 Department of Immunology, Genetics and Pathology, Section of Experimental and Clinical Oncology, Uppsala University Hospital, Uppsala, Sweden; Weill Cornell Medicine, UNITED STATES

## Abstract

**Background:**

Malignancies in the urinary tract and the kidney graft are quite common after kidney transplantation. In some selected cases tumours develop from donor-derived tissue.

**Objectives:**

We hypothesised that there is a clinical value to investigate donor/recipient origin in urologic malignancies in renal transplant recipients.

**Methods:**

In this retrospective study, including patients transplanted between the years 1969 and 2014 at Uppsala University Hospital, Sweden, 11 patients with malignancies in urinary tract and 4 patients with malignancies in kidney transplants were investigated. Donor/recipient origin of tumour tissue was analysed by polymerase chain reaction (PCR) of human leucocyte antigen (HLA) genotypes or by fluorescence in situ hybridization (FISH analysis) of sex chromosomes. HLA genotype and sex chromosomes of the tumour were compared to the known HLA genotype and sex chromosomes of recipient and donor.

**Results:**

Three of ten cancers in the urinary tract and three of four cancers in the kidney transplants were donor-derived.

**Conclusions:**

We suggest that urologic malignancies in renal transplant recipients can be investigated for transplant origin. In addition to conventional therapy the allograft immune response against these tumours can be valuable to treat donor-derived cancers.

## Introduction

Development of malignancies after organ transplantation is one of the major risks with lifelong immunosuppressive treatment. To assess the origin, malignant tumours after solid organ transplantation can be divided into de novo tumours, recurrence of earlier malignancies and donor-related malignant tumours. The donor-related tumours develop from donor tissue and can further be divided into donor-transmitted tumours and donor tissue-derived tumours [1]. While donor-transmitted tumours exist in the donor at the time of transplantation, the donor tissue-derived (donor-derived) tumours are malignancies that develop from donor tissue with no pre-existing malignancy in the donor and that consequently appear post-transplantation. Different types of donor-derived tumours have previously been described such as renal cell cancer [2], post-transplant lymphoproliferative disorders [3, 4], Kaposi’s sarcoma [5] and hepatocellular carcinoma [6]. These tumours occur quite rarely, having an estimated frequency of 0.01–0.05% [7–11].

Donor-derived tumours are managed by reduction or cessation of immunosuppression to allow the recipient’s own immune system to be activated. The tumours can be rejected merely by host immune surveillance. Most patients usually need conventional therapy such as surgery, chemotherapy or radiation [7, 9, 12, 13]. In addition to allograft immune responses these tumours offer new options for cancer treatment such as immunomodulatory or antiviral treatment options.

We hypothesised that there is a clinical value to investigate donor/recipient origin in urologic malignancies in renal transplant recipients. In this study we investigated whether urologic tumours in renal transplant recipients more frequently may be of donor origin to a greater degree than shown this far.

Hence, we investigated all cancers in the renal allograft or cancer of the urinary tract in transplanted patients between the years 1969 and 2014 with regard to tissue origin.

## Material and methods

### Identification of patients

Between June 1969 and December 2014, 2835 renal or simultaneous renal and pancreas transplantations were performed in 2437 patients at Uppsala University Hospital, Sweden. Ninety-two patients were lost to follow up, the remaining number of patients were 2345. All recipients received a kidney transplant or kidney and pancreas transplants according to the allocation rules of Scandiatransplant and according to Swedish legislation. None of the transplant donors was from a vulnerable population and all donors and the next of kin provided written informed consent that was freely given. Informed consent was obtained according to the Swedish law (National Board of Health and Welfare, SOSFS 2012:14) and according to Scandiatransplant allocation rules. The informed consent can, according to Swedish law, be found in a nationwide donor registry, be oral or written, or according to the interpretation of relatives to the donor. This is then signed by the treating physician.

**Fig 1:**
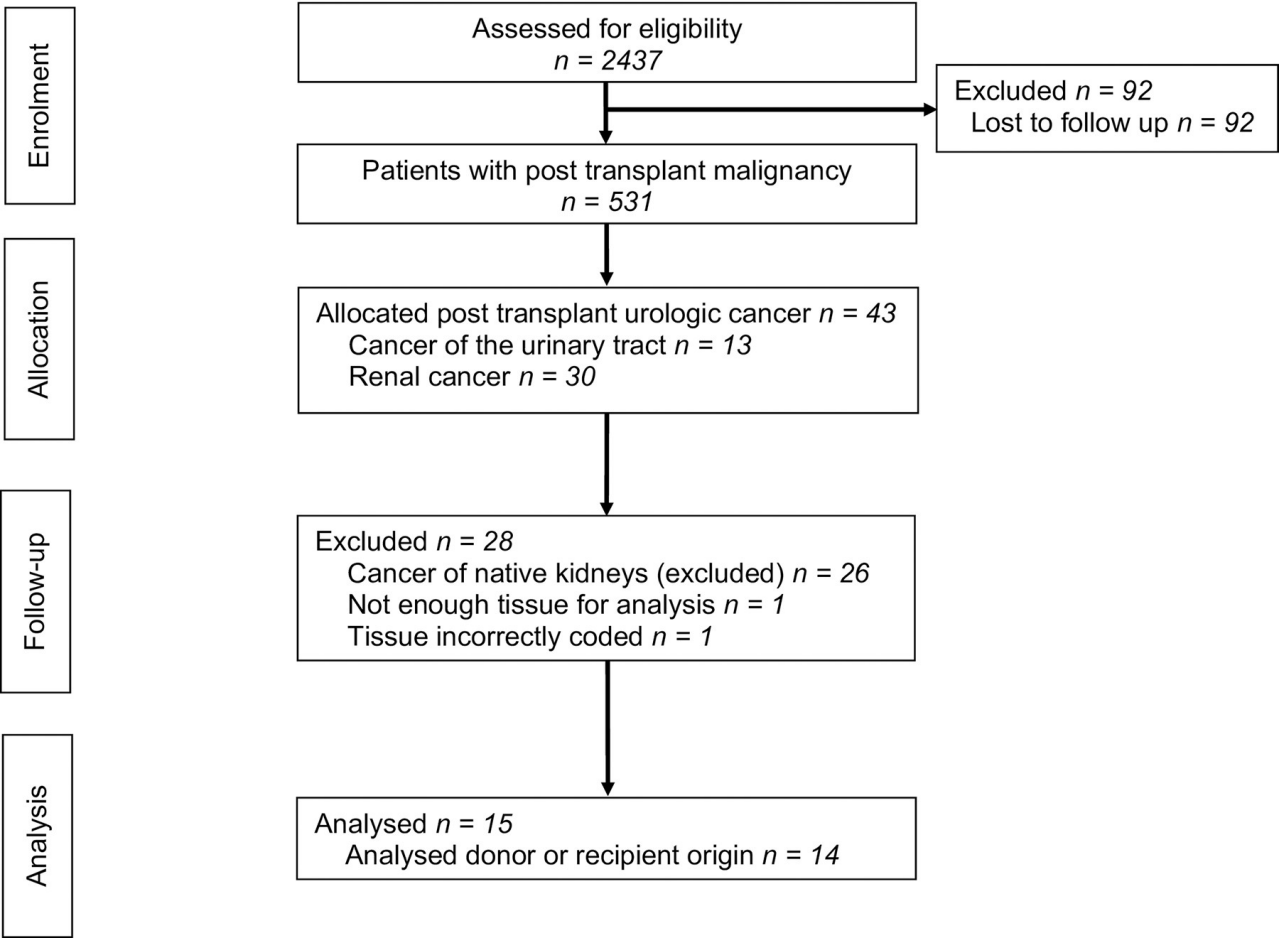
Flow diagram of donor-derived urologic cancers in renal transplanted patients.

Since September 2009 information on post-transplant malignancies have been retrieved by linkage between the Hospital Transplant Database and the Regional Tumour Register in the Uppsala-Örebro region (RTR). This allows for more than 95% detection of post-transplant malignancies [14]. In total 531 patients were diagnosed with post-transplant malignancies until August 2015. Forty-three patients were diagnosed with urologic malignancies, 13 patients with malignancy of the urinary tract and 30 patients with malignancy of the kidney. Of the 30 patients with malignancy in the kidney, 26 patients were found with tumour in the native kidney(s) and 4 patients with tumour in the kidney transplant (Fig 1).

Seventeen patients were included in this partly retrospective study; all patients with malignancy of the urinary tract (n = 13) as well as all patients with malignancy in the renal transplant (n = 4). Clinical data, including patient survival, were collected from medical records. Information on sex, age and HLA genotype of donor and recipient were retrieved from the hospital transplant database at the Department of Transplantation. The primary endpoint was to analyse the donor or recipient origin of the tumour.

Archival paraffin blocks of urologic tumour tissue were obtained from the respective regional hospitals of the patients. There was insufficient tumour tissue for further analysis in one patient, and accidently tumour tissue from another patient was not correctly coded; hence these tumours of the urinary tract (n = 2) were excluded from the analyses. Thus, the final number of included patients in the study was 15 (ClinicalTrials.gov identifier NCT02241564).

This study was approved by the Regional Ethical Review Board in Uppsala, Sweden (Dnr 2007/032) and approved by the Uppsala Biobank, Sweden (ID: BbA-827-2015-081). Written informed consent was obtained from all living patients prior to inclusion. The use of data and cancer tissue for analysis from deceased patients was approved by the Regional Ethical Review Board.

### Histology

An experienced pathologist re-evaluated the histology of the tumour tissue in the obtained archival paraffin blocks. Representative tumour material from transplantectomy specimens and biopsies from renal transplants and the urinary tract was fixed in 4% phosphate buffered formaldehyde and embedded in paraffin. Tissue sections (4 to 5 μm thick) were processed for routine histology and stained with haematoxylin-eosin, and Sirius red for collagen.

### Analysis of donor or recipient origin of tumours by HLA genotyping

HLA genotyping of the tumour was performed when the donor and recipient had different HLA-A, HLA–B or HLA-DRB1. Genomic DNA was extracted from manually dissected tumour tissue by using Recover All Total Nucleic Acid Isolation kit (Ambion, Austin, TX, USA) following the protocol of the manufacturer. The HLA region was amplified by polymerase chain reaction (PCR) using sequence-specific primers (SSP; Olerup, Stockholm, Sweden) according to a standard protocol [15]. HLA-A, HLA-B and HLA-DRB1 were routinely tested [16]. The HLA type in the tumour was compared to the known HLA profile of the recipient and the donor.

### Fluorescence in situ hybridization (FISH) analysis of different genders in tumours

FISH was used to determine donor or recipient origin of tumours in cases where the donor and recipient were HLA identical but had different gender. Representative tumour specimens, 4 μm thick, formalin-fixed and paraffin-embedded were analysed. First the tumour specimen was deparaffinised and incubated in hydrochloric acid (0.2M). After 20 min at room temperature the tumour specimen was rinsed and kept in VP2000 Pretreatment Reagent (Abbott Molecular Inc, Abbott Park, IL, USA). In order to separate the DNA strands a protease was added. DNA probes with fluorescent label, AneuVysion probe kit (Vysis CEP 18, X, Y-alpha satellite probe Abbott Molecular Inc), caused hybridization to the centromeres of the X- and Y-chromosomes. Vectashield mounting medium with 4’,6-diamidino-2-phenylindole (DAPI; Vector Laboratories Inc, Burlingame, CA, USA) was used to mount the chromosomes. To identify the XX or XY karyotype a fluorescence microscope (Zeiss, Oberkochen, Germany) was used. The analyses of the images were performed with the ISIS software (MetaSystems, Altlussheim, Germany).

### Statistics

All data were presented as medians with ranges. Occurrence of donor-derived tumours were presented as ratios.

## Results

### Patient characteristics

The characteristics of the patients are summarized in Table 1 and S1–S3 Tables. All study patients received a kidney transplant between 1981 and 2008. Eight patients received one renal transplant, five patients received two renal transplants, one of them had a simultaneous kidney and pancreas transplantation. Two patients received three renal transplants. Of all 24 renal allografts in these 15 patients 10 renal allografts originated from living donors and 14 renal allografts originated from deceased donors. The median age at transplantation was 43 years (range 22 to 64 years). Median duration from first transplantation to cancer diagnosis was 14 years (ranged 2 to 23 years). The gender distribution women: men was 1:2.

**Table 1:** Patient characteristics.

	Cancer in the kidney transplant	Cancer in the urinary tract
Number of patients	4	11
Number of kidney transplants	6	18
Age at 1:st tx, (years)	34 (22–47)	48 (28–64)
Donor age, (years) ^1^	42.5 (40–59)	54.5 (17–73)
Type of tx LD/DD^1^	4/2	6/12
Duration 1:st tx to ca, (years)	16 (6–24)	10 (2–22)
Age at ca dg, (years)	47 (37–71)	62 (45–71)
Gender recipient (female/male)	2/2	3/7
Gender donor (female/male) ^1^	1/5	7/11
Cancer derived (donor/recipient)^2^	3/4	3/10 ^2^
Creatinine at ca dg, median (range)	163 (157–210)	187 (96–260)
Number of pat in dialysis at ca dg	1	2
Rejection treatment (percent)	0%	55% ^3^

Tx = transplantation, ca = cancer, dg = diagnosis.

^1^ = retransplantations; two retransplantations among patients with cancer in the kidney transplant and two retransplantations among patients with cancer in the urinary tract.

^2^ = HLA identical donors of same gender in one patient

^3^ = no available information of rejections in one patient.

Age at first transplantation, duration first transplantation to cancer diagnosis, age cancer diagnosis donor age and creatinine are presented as median with ranges.

The immunosuppressive protocol was in concordance with the maintenance immunosuppression used at the Department of Surgery, Section of Transplantation Surgery in Uppsala at the time the patients were transplanted (intention to treat). All patients who were transplanted before the year 2000 (n = 11) received a T-cell depleting agent as induction theraphy while all patinets transplanted from the year 2000 and forth (n = 7) received an anti-IL2 receptor antagonist as induction therapy. Maintenance immunosuppression is mentioned in S4–S6 Tables for each patient. Acute rejections were treated with corticosteroids alone (n = 3), corticosteroids and anti-thymocyte globulin (n = 1) or corticosteroids and plasmapheresis (n = 1) (S4–S6 Tables).

### Tumour characteristics

Of the 11 patients with cancer of the urinary tract, 9 patients suffered from cancer of the bladder and 2 patients experienced cancer of the ureter. Four patients were diagnosed with cancer of the renal allografts. The morphological diagnoses of the cancers in the bladder were distributed between urothelial cancer (n = 8) and adenocarcinoma (n = 1), while the cancers of the transplant ureter comprised urothelial cancer (n = 2). The morphological diagnoses of the renal cancers were clear cell carcinoma (n = 1), adenocarcinoma (n = 1) and papillary cancer (n = 2) (S4–S6 Tables). The patient with adenocarcinoma in the renal transplant died a few years later from advanced rectal cancer.

### Origin of the tumour

The results of the HLA genotyping and FISH analyses showed that 6 of 14 (43%) examined tumours were of donor origin, 3 of 10 tumours (30%) in the urinary tract and 3 of 4 tumours (75%) in the renal allografts (S1–S3 Tables). One of the cancers in the renal allografts was identified to have tissue of recipient origin and another tumour was identified to have cells/tissue of two tissue types, i.e. cells/tissue of donor and recipient origin. In both cases the tumour tissue was extracted from renal allografts after transplantectomy. One male patient received renal allografts from live donors twice, both times from HLA identical brothers, and therefore the origin of tumour could not be analysed. The cancers developed 14 years (range 2 to 23 years) after the corresponding transplantation.

### Treatment and outcome of the cancers

Treatment and outcomes are presented in data in S1 Text.

## Discussion

This survey showed that three of four investigated cancers of the renal allografts and three of ten examined cancer of the urinary tract originated from the donor. One of the cancers in the renal allograft was identified to have cells/tissue of two tissue types, i.e. cells/tissue of donor and recipient origin. An explanation for mixed tissue origin could be that the tumour is of donor origin and invading lymphocytes of recipient origin.

At least 0.2% (6 of 2345 patients) of all transplanted patients in Uppsala and 1% (6 of 531 patients) of all patients with post-transplant malignancies developed donor-derived tumours. No donor-transmitted tumours are included in these numbers. Most current studies comprise only donor-transmitted (0.01–0.05%) and not donor-derived malignancies because the latter has not been systematically investigated [7, 8]. Donor-derived tumours probably occur more commonly than shown so far. Five of 8 Kaposi’s sarcomas [5] originated from the donor and 16 of 43 post-transplant lymphoproliferative disease (PTLD) [3] were shown to be of donor origin. Most of those donor derived PTLD:s were however located in the renal transplant. In a later study on PTLDs all tumours in 67 patients were of recipient origin [15].

One of the cancers in the renal allografts originated from the recipient, and three were of donor origin. Boix et al. observed the same phenomenon in a kidney transplant 2009 [16]. They hypothesized that recipient stem-cells or circulating malignant transformed cells could contribute to the recipient-derived cancer in the graft [16]. In addition, LaBerge et al. suggested cell hybridization as explanation to a donor-patient hybrid genome found in a melanoma lymph node metastasis after bone marrow transplantation [17]. Donor-derived stem-cells have been identified and suggested to contribute to the progression of squamous cell carcinoma of the skin in renal transplanted patients [18].

Cancers of the kidney and urinary tract occur at a 6-fold and 2-fold increased incidence respectively among renal transplanted patients compared to immunocompetent individuals in Sweden [19]. The standardized mortality ratio (SMR) due to kidney cancer was 4.4 and the SMR due to cancer of the urinary tract was 4.7 compared to the general Chinese population [20]. In Asia post-transplant kidney cancer and cancer of the urinary tract occur more commonly than in Europe and USA.

Donor-derived tumours compose a unique immunological feature because they occur as a consequence of immunosuppressive treatment after organ or cell transplantation [21]. This offers new treatment opportunities because the host allograft response may be directed against the cancer.

The strengths of the study were that all patients at the department of transplantation in Uppsala diagnosed with cancer in the kidney transplant or in the bladder were included. Clinical information was confirmed in the medical records and all analyses were performed at the same department of pathology. The number of patients was however limited and therefore the estimated proportion of donor-derived patients may change in a larger patient material. The proportion of the results might also have been influenced by the prolonged storage time of some tumour specimens; 2 of 15 tumour specimens were indeterminable.

In conclusion, we suggest that donor-derived tumours may occur more frequently than previously thought. We suggest that urologic malignancies in renal transplant recipients are investigated for donor/recipient origin. Donor-derived tumours are unique from an immunological point of view and this offers new options for cancer treatment by immunomodulatory drugs in addition to reduced immunosuppression.

## Supporting information

S1 Checklist TREND statement checklist. (PDF)

S1 Table Characteristics of recipients and donors in patients with cancer in the kidney transplant. (PDF)

S2 Table Characteristics of recipients and donors in patients with verified transplant-derived cancers in the urinary tract. (PDF)

S3 Table Characteristics of the patients with recipient-derived cancer of the urinary tract. (PDF)

S4 Table Characteristics of cancers in kidney transplants. Treatment and outcome.
(PDF)

S5 Table Characteristics of donor derived cancers in the urinary tract. Treatment and outcome.
(PDF)

S6 Table Characteristics of recipeint derived cancers in the urinary tract. Treatment and outcome.
(PDF)

S1 Text Treatment and outcome of the urological cancers. (PDF)

S1 File Documentation Donor (English). (PDF)

S2 File Documentation Donor (Swedish). (PDF)

S3 File (DOC)

## Abbreviations

DD: deceased donor
FISH analysis: fluorescence in situ hybridization
HLA: human leucocyte antigen
LD: living donor
mTOR inhibitor: inhibitor of mammalian target of Rapamycin
PTLD: post-transplant lymphoproliferative disease
