# Supplementary material for: Donor-derived urologic cancers after renal transplantation: A retrospective non-randomized scientific analysis
Source: PLoS One. 2022 Sep 21;17(9):e0271293. doi: 10.1371/journal.pone.0271293 (PMC9491581; doi:10.1371/journal.pone.0271293)
Supplement: S1 Table — (PDF) [file pone.0271293.s002.pdf]

S1 Table. Characteristics of recipients and donors in patients with cancer in the kidney transplant.

**Patient**	**1**	**2**	**3**	**4**
Renal disease	IgA	PN	GN	Cyst, (DM)
Month,Year(s) of tx	Aug 1993, Jan 2008	Sept 1989	Mars 1988	Jul 2000, Jul 2014
Age at 1:st tx (years)	27	31	47	22
Tx with donor derived ca	1993	1989	1989	2000
Age at tx with donor derived ca	27	31	47	22
Donor age	40	59	41	46
Type of tx	DD,LD	LD	LD	LD,DD
Time tx to donor derived ca (years)	16	21	23	14
Age at ca dg (years)	43	52	70	36
Recipient gender	M	M	F	F
Donor gender	M	M	M	F
Donor/recipient derived	Donor	Recipient	Donor	Donor/Recipient
HLA type recipient	A3,24, B7,47<br>DR15, DQ 06	A3, B 14,35<br>DR 5,6	A2, B7,60<br>DR 01,04	A1,2, B7,8<br>DR03, 14
HLA type donor I	A2,3, B12,17<br>DR 04,07<br>DQ02,03	A3, B35<br>DR1,5	A2,28, B7,40<br>DR01,04	A2,32, B7,61<br>DR01,14
HLA type donor II				A2, B37,44<br>DR1 04,07<br>DQ 02
HLA type cancer	A2,3, B12,17<br>DR 04,07<br>DQ02,03	A3, B 14,35<br>DR 5,6	A2,28, B7,40<br>DR01,04	A2,32, B7,61<br>DR01,14<br>A1,2, B7,8<br>DR03, 14
Creatinin post ca dg	157	210	Dialysis	163
Creatinin 1 year post ca dg	157	280	Dialysis	146
Rejection	No	No	No	No
Treatment of rejection	No	No	No	No

Tx = transplantation, ca = cancer, dg = diagnosis, GN = glomerolonephritis, Cyst = polycystic kidney disease, DM = diabetes mellitus, GS = glomerulosclerosis, IgA = IgA-nephritis, PN = pyelonephritis, P-tx = pancreas transplanted, M = male, F = female.
