# Supplementary material for: Donor-derived urologic cancers after renal transplantation: A retrospective non-randomized scientific analysis
Source: PLoS One. 2022 Sep 21;17(9):e0271293. doi: 10.1371/journal.pone.0271293 (PMC9491581; doi:10.1371/journal.pone.0271293)
Supplement: S2 Table — (PDF) [file pone.0271293.s003.pdf]

**S2 Table. Characteristics of recipients and donors in patients with verified donor-derived cancers in the urinary tract.**

| **Patient** | **5** | **6** | **7** |
|---|---|---|---|
| Renal disease | GN | GS | GN |
| Month, Year(s) of tx | Jan 1990, Jun 1998 | Jun 1993, Jan 2000 | Mar 1989, Jan 1990, Aug 2006 |
| Age at 1:st tx (years) | 39 | 48 | 40 |
| Time 1:st tx to ca (years) | 16 | 14 | 22 |
| Tx with donor derived ca | 1989 | 1993 | 2006 |
| Age at tx with donor derived ca | 39 | 48 | 57 |
| Donor age | 58 | 73 | 56 |
| Type of tx | DDx2 | DDx2 | DDx3 |
| Time tx to donor derived ca (years) | 16 | 14 | 5 |
| Age at ca dg (years) | 55 | 62 | 62 |
| Recipient gender | M | M | F |
| Donor gender | M | F | M |
| Donor/recipient derived | Donor | Donor | Donor |
| HLA type recipient | A2, B7<br>DR 07,15<br>DQ 03,06 | A2, B12,15<br>DR1,4 | A2, B27,44<br>DR 01,15<br>DQ 05,06 |
| HLA type donor and cancer | A1,3, B18,8<br>DR2,3 | A2, B12,15<br>DR4 | A1,2, B37,5<br>DR01,13<br>DQ 05,06 |
| Creatinin post ca dg | Dialysis | 174 | 96 |
| Creatinin 1 year post ca dg | Dialysis | | 96 |
| Rejection | No | No | No |
| Treatment of rejection | No | No | No |

Tx = transplantation, ca = cancer, dg = diagnosis, GN = glomerolonephritis, GS = glomerulosclerosis, M = male, F = female.
