# Supplementary material for: Donor-derived urologic cancers after renal transplantation: A retrospective non-randomized scientific analysis
Source: PLoS One. 2022 Sep 21;17(9):e0271293. doi: 10.1371/journal.pone.0271293 (PMC9491581; doi:10.1371/journal.pone.0271293)
Supplement: S3 Table — (PDF) [file pone.0271293.s004.pdf]

S3 Table. Characteristics of the patients with recipient-derived cancer of the urinary tract.

**Patient**	**8**	**9**	**10**	**11**	**12**	**13**	**14**	**15**
Renal disease	PN	Wegener, DM	Trauma	Cyst	Reflux	Cyst	Cyst	IgA nephritis
Month, Year(s) of tx	Apr 1988	Febr 1996	Oct 1997	Jan 1985, Aug 1985, Jun 1993	Jun 1981, Nov 1993	Jul 2004	Dec 2001	Nov 2005
Age at 1:st tx (y)	57	61	43	53, 61	28, 40	64	60	41
Donor age	17	52	57	19, 52, 59	32, 44	56	35	37
Time 1:st tx to ca (y)	2	2	3	16	21	6	10	10
Type of tx	DD	LD	DD	DD, LDx2	LDx2	DD	DD	LD
Age at ca dg (y)	59	63	45	69	49	70	71	51
Recipient gender	F	M	F	M	M	M	M	M
Donor gender	M	F	M	M, M, F	M,M	M	F	F
Tumour origin	Recipient	Recipient	Recipient	Recipient	Unknown	Recipient	Recipient	Recipient
HLA type recipient	A1,19 B8 DR3	A2,3 B60,47 DR4,13	A1,2 B51,41 DR7,13	A2,19 B 12,15 Dr 6	A9,11 B5,12 Cw4 DR 15	A1,24 B8,13, DRB1 03,07 DQBI 02	A2 B7,60 DRB1 15,04	A2,29(19) B7,3 DRB1 12,13 DQB1 03,06
HLA type 1:st donor	A9,25 B18,40 DR not analysed	A1,2 B44,57 DR 1,4	A1,2 B51,8 DR3,7	A9,11 B5,15 DR15,13	HLA identical	A11,28 B51,62, DRB1 07,13 DQB1 02,06	A1,2 B17,27 DR04,07	A3,11 B8,35, DRB1 03,13 DRQ1 02,06
HLA type 2:nd donor				A2,26, B5,12 CW1, DR1,7	HLA identical			
HLA type 3:rd donor				HLA identical				
Creatinin post ca dg	>260	155	113	200	120	250	200	Dialysis
Creatinin 1 y post ca		130		247			200	
Rejection	Unknown	No	Yes	Yes	No	Yes	Yes	Yes
Treatment of rejection	Unknown	No	Cs	Cs, ATG	No	Cs	PF, Cs	Cs

Tx = transplantation, ca = cancer, dg = diagnosis, y = years, Cyst = polycystic kidney disease, DM = diabetes mellitus, Reflux = ureter reflux, IgA = IgA-nephritis, PN = pyelonephritis, Wegener = Wegeners granumalotosis, M = male, F = female, Cs = cortocosteroids, ATG = Anti-thymo globuline, PF = plasmaferesis.
