# Supplementary material for: Donor-derived urologic cancers after renal transplantation: A retrospective non-randomized scientific analysis
Source: PLoS One. 2022 Sep 21;17(9):e0271293. doi: 10.1371/journal.pone.0271293 (PMC9491581; doi:10.1371/journal.pone.0271293)
Supplement: S4 Table — Treatment and outcome. (PDF) [file pone.0271293.s005.pdf]

S4. Table. Characteristics of cancers in the kidney transplants. Treatment and outcome.

**Patient**	**1**	**2**	**3**	**4**
Dg ICD 10	C64	C64	C64	C64
Localisation of cancer	Renal tx	Renal tx	Renal tx	Renal tx
Histology of tumour	Clear cell ca	Adenoca	Adenoca	Papillary ca
TNM classification (WHO 2009)	T1N0M0	T1N0M0	T1N0M0	T1N0M0
Histological grading	Fuhrman 1	Highly diff	Highly diff	Highly diff
Clinical grading	Low risk	Low risk	Interm risk	Low risk
Treatment	Tx-tomy<br>mTOR	RF, Tx-tomy<br>mTOR	Tx-tomy<br>No IS	Tx-tomy
BKV positive tumour	Neg	Neg	Neg	Neg
BK viremin	Unknown	No	Unknown	No
BKV transplant nephritis	No	No	No	Unknown
BKV treatment				
Earlier IS	CyA, Cs	CyA, Cs	Aza, Cs	Tac, Aza, Cs
IS at ca dg	Tac, MMF, Cs	CyA, Cs	CyA, Cs	Tac, MMF, Cs
IS after ca dg	mTOR, Cs	CyA	None	Tac, MMF, Cs
Treatment result after 6 months	CR	CR	CR	CR
Treatment result at 1 year	CR	CR	De novo ca	CR
Treatment result at 2 year	CR	Recurrent ca	De novo ca	
Time ca dg to death (months)				
Cancer induced death				

Dg ICD 10 = Diagnosis according to International Classification of Diagnosis version 2010, IS= immunosuppression, dg = diagnosis, ca = cancer, tx= transplant, Tx-tomy = transplantectomy, Pos =positive, Neg = negative, highly diff = highy differentiated, Interm risk = intermediate risk, RF = Radiofrequency ablation, CR = complete remission, IS = immunosuppression, Aza = Azathioprine,corticosteroids, MMF = Mycophenolate mofetil, CyA = Cyclosporin, Tac = Tacrolimus, Cs = corticosteroids, mTOR = mTOR inhibitors.
