# Supplementary material for: Donor-derived urologic cancers after renal transplantation: A retrospective non-randomized scientific analysis
Source: PLoS One. 2022 Sep 21;17(9):e0271293. doi: 10.1371/journal.pone.0271293 (PMC9491581; doi:10.1371/journal.pone.0271293)
Supplement: S5 Table — Treatment and outcome. (PDF) [file pone.0271293.s006.pdf]

S5. Table. Characteristics of donor-derived cancers in the urinary tract. Treatment and outcome.

**Patient**	**5**	**6**	**7**
Dg ICD 10	C67	C66	C67
Localisation of cancer	Bladder	Ureter	Bladder
Histology of tumour	Adenoca	Urothelial ca	Urothelial ca
TNM classification (WHO 2009)	T2NXMX	T4bG3N3M1	T2bN2M0
Histological grading	G2	G3	G3
Clinical grading	High risk	High risk	High risk
Treatment	TUR-B Tx-tomy,	Tx-tomy CD40	Cidofovir, mTOR, RT
BKV positive tumour	Pos	Neg	Pos
BK viremin	Unknown	Unknown	Yes
BKV transplant nephritis	Unknown	Unknown	Yes
BKV treatment			Cidofovir
Earlier IS	Aza, CyA, Cs	CyA, Aza, Cs	CyA, Aza, Cs
IS at ca dg	CyA, Cs	CyA,Cs	Tac, MMF, Cs
IS after ca dg	None	Cs	mTOR, Cs
Treatment result after 6 months	Regress	Regress	Regress
Treatment result at 1 year	CR	Dead	Progress
Treatment result at 2 year	CR		Dead
Time ca dg to death (months)		10	22
Cancer induced death		Yes	Yes

Dg ICD 10 = Diagnosis according to International Classification of Diagnosis version 2010, IS= immunosuppression, dg = diagnosis, ca = cancer, tx= transplant, Tx-tomy = transplantectomy, Pos =positive, Neg = negative, TUR-B = transurethral resection of the bladder, CR = complete remission, Aza = Azathioprine,corticosteroids, MMF = Mycophenolate mofetil, CyA = Cyclosporin, Tac = Tacrolimus, Cs = corticosteroids, mTOR = mTOR inhibitors, RF = Radiofrequency ablation.
