# Supplementary material for: Donor-derived urologic cancers after renal transplantation: A retrospective non-randomized scientific analysis
Source: PLoS One. 2022 Sep 21;17(9):e0271293. doi: 10.1371/journal.pone.0271293 (PMC9491581; doi:10.1371/journal.pone.0271293)
Supplement: S6 Table — Treatment and outcome. (PDF) [file pone.0271293.s007.pdf]

S6 Table. Characteristics of recipient-derived cancers in the urinary tract. Treatment and outcome.

**Patient**	**8**	**9**	**10**	**11**	**12**	**13**	**14**	**15**
Dg ICD 10	C67	C67	C67	C67	C67	C66	C67	C67
Localisation of cancer	Bladder	Bladder	Bladder	Bladder	Bladder	Ureter	Bladder	Bladder
Histology of tumour	Urothelial ca	Urothelial ca	Adenoca	Urothelial ca	Urothelial ca	Urothelial ca	Urothelial ca	Urothelial ca
TNM classification (WHO 2009)	T4NXMX	T1N0M0G3	T3bNXM0	T in situ	T2bNXMX	T4NXMX	T1N0MX	T1N0M0
Histological grading	Unknown	G3	G3	G3	G3	Unknown	G3	G3
Clinical grading	High risk	Interm risk	High risk	Interm risk	High risk	High risk	Interm risk	Interm risk
Treatment	Inoperable	PVC Bricker	Cystectomy, RT, ChemoT	TUR-B	Inoperable	Inoperable	TUR-B Mitomycin	TUR-B, Mitomycin, BCG No IS
BKV positive tumour	Neg	Neg	Neg	Neg	Neg	Pos	Pos	Pos
BK viremin	Unknown	Unknown	Unknown	Unknown	Unknown	Yes	Yes	Yes
BKV transplant nephritis	Unknown	Unknown	Unknown	Unknown	Unknown	Unknown	Yes	Yes
BKV treatment						IS decreased	Cidofovir	IS decreased
Earlier IS	CyA, Aza, Cs	CyA, Cs	CyA, MMF, Cs	CyA, Cs	Aza, Cs	Tac, MMF, Cs	Tac, MMF, Cs	Tac, MMF, Cs
IS at ca dg	CyA, Aza, Cs	CyA, Cs	CyA, MMF, Cs	CyA, MMF	CyA, Cs	Tac, Cs	Tac, Cs	Tac, Cs
IS after ca dg		CyA, Cs	CyA, MMF, Cs	CyA	CyA, Cs	Tac, Cs	mTOR, Cs	No IS
Treatment result after 6 months	Dead	Recurrent	Recurrent	Recurrent	Dead	Dead	Recurrent	CR
Treatment result at 1 year		CR	Dead	CR			CR	
Treatment result at 2 year		CR		De novo ca			CR	
Time ca dg to death (months)	2	50	5	48	0.2	6		
Cancer induced death	Yes	No	Yes	De novo ca	Yes	Yes		

Dg ICD 10 = Diagnosis according to International Classification of Diagnosis version 2010, IS= immunosuppression, dg = diagnosis, ca = cancer, tx= transplant, Tx-tomy = transplantectomy, Pos =positive, Neg = negative, G = grade, Interm risk = intermediate risk, TUR-B = transurethral resection of the bladder, CR = complete remission, Aza = Azathioprine,corticosteroids, MMF = Mycophenolate mofetil, CyA = Cyclosporin, Tac = Tacrolimus, Cs = corticosteroids, mTOR = mTOR inhibitors, CPV = cystoprostatovesiculectomy, Bricker = Bricker´s deviation, BCG = BCG instillation, RT = radiation therapy, Chemo T= Chemotherapy, M-mycin = Mitomycin instillation.
