# Supplementary material for: Donor-derived urologic cancers after renal transplantation: A retrospective non-randomized scientific analysis
Source: PLoS One. 2022 Sep 21;17(9):e0271293. doi: 10.1371/journal.pone.0271293 (PMC9491581; doi:10.1371/journal.pone.0271293)
Supplement: S1 Text — (PDF) [file pone.0271293.s008.pdf]

S7 Text. Treatment and outcome of the urological cancers.

Treatment and outcome of patients with cancer in the ureter

Both patients with cancers of the ureter had inoperable advanced cancers (stage T4) with metastases in the bladder at the time of diagnosis. One of them died shortly after diagnosis.

When the second patient with cancer in the ureter was referred to the Uppsala University hospital a clinical trial was on-going that used immunostimulating gene therapy consisting of an adenoviral vector that transferred the gene for human immunostimulator CD40 ligand (AdCD40L) on patients with high-grade urinary bladder cancer. A ureter-transplantectomy with removal of the primary tumour of donor origin was performed. Immunosuppressive treatment was reduced to a low dose of steroids. The patient received ultrasound-guided injections containing 2.5 x 10^{11} AdCD40L vector particles in metastases once a week, in total 24 injections during a time span of 37 weeks. The patient regained most of the former health and experienced a good quality of life a few weeks after the start of the treatment. The cancer regressed or remained stable for eight months. At this time, the general condition of the patient worsened due to tumour progression. The antibody titres against the vector, which previously had been on a low level, had risen. A few weeks later the patient died.

Treatment and outcome of cancer in the bladder stage T1

All four patients with bladder cancer stage T1 were treated with transurethral resection. Two of them also received local Mitomycin instillations and one of them further received BCG instillations in the bladder. All four of them experienced recurrent cancer within a year; three of them underwent another transurethral resection, while the fourth

patient underwent surgery with cystoprostatovesiculectomy and Bricker deviation. No recurrent cancers have been diagnosed during the follow up.

Treatment and outcome of cancer in the bladder stage T>1

The surgical treatment in five patients with cancer of the bladder (stage T>1) consisted of cystoprostatovesiculectomy, radiation therapy and chemotherapy (n=1), transurethral resection (n=1) and transurethral resection in combination with transplantectomy (n=1). For the remaining patients the cancer was advanced beyond surgical treatment (n=2). Two of these five patients survived less than 6 months.

One patient initially underwent transurethral resection of an adenocarcinoma of the urinary bladder. A year later bilateral transplantectomy was performed. Macroscopically no tumour was found, but microscopically the cancer was detected in the resection margins of the transplant ureters bilaterally. Immunosuppressive treatment was interrupted and further therapy, such as radiation and possibly chemotherapy, was planned; however, the patient never received the planned treatment. Nevertheless, three years after the original malignancy diagnosis, he was in a good clinical condition and without signs of disseminated disease as shown by magnetic resonance imaging and cystourethroscopy. The patient is alive today 13 years after the diagnosis of the first donor-derived bladder cancer.

Another patient developed obstruction of the transplant ureter. A low differentiated urothelial cancer with diffuse growth in the wall of the bladder as well as in the transplant ureter was diagnosed by cystourethroscopy. Metastases in local lymph nodes in the pelvis region were found on CT scan and PET scan. HLA genotyping confirmed that the cancer was of donator origin from the third renal transplant still functioning. All tumours cells, but not the surrounding normal cells were strongly

positive for SV40 T-antigen. The cancer was advanced beyond surgical treatment and the recommendation was to interrupt the immunosuppressive treatment so that the patient could reject the tumour. However, the patient refused that treatment option. Therefore, her immunosuppression was minimized and switched to an mTOR inhibitor. She also received antiviral treatment (Cidofovir 0.5 mg/kg) once a week for 21 weeks.

The clinical condition of the patient improved. The cancer regressed and after a few weeks the urine could pass through the transplant ureter. After 12 weeks no metabolic activity could be observed on positron emission tomography and no tumour mass or enlarged lymph nodes were detected by computer tomography. Fifteen months after diagnosis the cancer progressed and 7 months later the patient died due to peritoneal carcinomatosis.

Treatment and outcome of patients with cancer in the renal allografts

The cancers in the four renal allografts were small in size ranging from 1 to 2 cm in diameter. One patient was treated with radiofrequency ablation. However, a transplantectomy was performed two years later due to recurrent cancer in combination with progressing renal failure. The three remaining transplants with cancers were extirpated; all of the transplants were non-functioning.

The immunosuppression was modified after cancer surgery in three of four patients as follows. The immunosuppression was switched to mTOR inhibitor (n=2). Due to adverse effects (fatigue and skin rash) it was changed back to the original immunosuppression in one patient. Immunosuppression was interrupted (n=1) or continued without any further changes (n=1) due to later retransplantation.
