# Supplementary material for: Donor-derived urologic cancers after renal transplantation: A retrospective non-randomized scientific analysis
Source: PLoS One. 2022 Sep 21;17(9):e0271293. doi: 10.1371/journal.pone.0271293 (PMC9491581; doi:10.1371/journal.pone.0271293)
Supplement: S1 File — (PDF) [file pone.0271293.s009.pdf]

Machine Translated by Google

# Record document for donation intervention on deceased donor (DBD / DCD) SOSFS 2012:14

**Points 1-6 are filled in by the doctor (s) responsible for the care of the donor**

**1.** Name, social security number: ______

**2.** Information on the investigation of willingness to donate

The will is **known** ☐ Donationsregistret ☐ Orally ☐ Written / digital ______

The will is **interpreted** ☐ Relatives have interpreted the will as positive

The will is **unknown** ☐ Investigation shows no reason to assume that the procedure is against the will of the individual and relatives have not vetoed **3. Donation of organs and tissues applies to:**

☐ Transplantation and other medical purposes ☐ For transplantation only

The donation applies with the exception of the following organs / tissues: ______

**4.** Related parties informed: ______

**5.** Cause of death (diagnosis): ______

**6.** Death determined (date & time): ______

**The doctor (s) who decided that the donation procedure may be performed:**

| *date* | *Hospital + Department* | *Signature + name clarification* |
|---|---|---|
| | | |

**Points 7-11 are filled in by the transplant surgeon who is responsible for the donation procedure**

Date and time for op.start ______

**7.** Data checked:

☐ Identity checked against ID tape

☐ Protocol for Determining Human Death Using Direct Criteria (DBD)

☐ Death certificate (indirect criteria) (DCD)

☐ Journal document for donation procedures for the deceased - this document, questions 1-6

☐ Blood grouping ☐ Donor characterization ☐ virusesvar

**8. Organs and tissues recovered for transplantation:**

ÿ **Liver** YJA10 ÿ **Kidneys** YKA02 ÿ **Pancreas** for whole transplant YJD30 ÿ **Pancreas** for islet cell production YJD00

ÿ **Heart** for transplant YFA00 ÿ **Lungs** YGA00 ÿ **Heart** for homograph YFA50

ÿ **Spleen** for immunological analysis ÿ **Vascular graft:** ______ ÿ Other organ / tissue ______

**9.** Organs and tissues used for other medical purposes (research):

ÿ **Lever** ÿ **Kidney / kidneys** ÿ **Pancreas**

ÿ **Heart** ÿ **Lung / lungs** ÿ Other organ / tissue ______

**10.** Reason why the donation procedure was not completed:

**11.** Findings of significance for autopsy or forensic examination: ÿ **No** ***(For forensic examination, any findings are described in a separate medical record / operation report)*** ÿ **Yes**

**Doctors who performed the donation procedure:**

| *date* | *Transplant unit* | *Signature + name clarification* |
|---|---|---|
| | | |

Scandinavian number:
