# Supplementary material for: Donor-derived urologic cancers after renal transplantation: A retrospective non-randomized scientific analysis
Source: PLoS One. 2022 Sep 21;17(9):e0271293. doi: 10.1371/journal.pone.0271293 (PMC9491581; doi:10.1371/journal.pone.0271293)
Supplement: S2 File — (PDF) [file pone.0271293.s010.pdf]

Journalhandling för donationsingrepp på avliden donator (DBD/DCD)
SOSFS 2012:14

Punkterna 1-6 fylls i av den/de läkare som ansvarar för vården av donatorn

1. Namn, personnummer: ______________________

2. Uppgifter om utredning av donationsvilja

Viljan är **känd** ☐ Donationsregistret ☐ Muntligt ☐ Skriftligt/digitalt ______________

Viljan är **tolkad** ☐ Närstående har tolkat viljan som positiv

Viljan är **okänd** ☐ Utredning visar ingen anledning att anta att ingreppet strider mot den enskildes vilja och anhöriga har inte lagt in sitt veto

3. Donation av organ och vävnader gäller för:

☐ Transplantation och annat medicinskt ändamål ☐ Enbart för transplantation

Donationen gäller med undantag av följande organ/vävnader: ______________

4. Närstående som informerats: ______________________

5. Dödsorsak(diagnos): ______________________

6. Dödsfallet fastställt (datum & klockslag): ______________________

Den/de läkare som beslutat om att donationsingreppet får utföras:

_______________	_______________	_______________
Datum	*Sjukhus + Avdelning*	*Namnteckning + namnförtydligande*

Punkterna 7-11 fylls i av den transplantationskirurg som ansvarar för donationsingreppet

Datum och tid för op.start ______________________

7. Uppgifter som är kontrollerade:

☐ Identitet kontrollerad mot ID-band

☐ Protokoll för bestämmande av människans död med hjälp av direkta kriterier (DBD)

☐ Dödsbevis (indirekta kriterier) (DCD)

☐ Journalhandling för donationsingrepp på avliden - detta dokument, frågor 1-6

☐ Blodgruppering ☐ Donatorskarakterisering ☐ Virussvar

8. Organ och vävnader som tagits tillvara för transplantation:

☐ **Lever** YJA10 ☐ **Njurar** YKA02 ☐ **Pancreas** för heltransplantation YJD30 ☐ **Pancreas** för ö-cellsframställning YJD00

☐ **Hjärta** för transplantation YFA00 ☐ **Lungor** YGA00 ☐ **Hjärta** för homograf YFA50

☐ **Mjälte** för immunologisk analys ☐ **Kärlgraft:** ______________ ☐ Annat organ/vävnad ______________

9. Organ och vävnader som tagits tillvara för annat medicinskt ändamål (forskning):

☐ **Lever** ☐ **Njure/njurar** ☐ **Pancreas**

☐ **Hjärta** ☐ **Lunga/lungor** ☐ Annat organ/vävnad ______________

10. Orsak till att donationsingreppet inte fullföljdes:

11. Fynd av betydelse för obduktion eller rättsmedicinsk undersökning: ☐ **Nej** ☐ **Ja**

(Vid rättsmedicinsk undersökning beskrivs eventuella fynd i separat journalhandling/operationsberättelse)

Läkare som utfört donationsingreppet:

_______________	_______________	_______________
Datum	*Transplantationsenhet*	*Namnteckning + namnförtydligande*

Scandianummer:
