# Supplementary material for: Donor-derived urologic cancers after renal transplantation: A retrospective non-randomized scientific analysis
Source: PLoS One. 2022 Sep 21;17(9):e0271293. doi: 10.1371/journal.pone.0271293 (PMC9491581; doi:10.1371/journal.pone.0271293)
Supplement: S3 File — (DOC) [file pone.0271293.s011.doc]

# Protocol

**MALTX**

Title: Projekt för uppföljning av MALignitet hos transplanterade (TX)patienter

| Authors | Vivan Hellström |
| --- | --- |
| Principal Investigator | Gunnar Tufveson |
| Steering Committee | Vivan Hellström, Gunnar Tufveson Dept of Transplantation, Hans Hagberg, Gunilla Enblad Dept of Oncology, Akademiska sjukhuset Uppsala. Erland Olausson Dept of Medicine, Sundsvalls sjukhus Emöke Dimeny, Dept of Medicine,Torgny Rasmuson Dept of Oncology, Norrlands universitetssjukhus Umeå, Lars Wennberg/Henryk Wilczek Dept of Transplantation, Karolinska University Hospital, Huddinge. Jana Roussos Dept of Transplantation, Sahlgrenska University Hospital of Gothenburg. |
| Document type | Clinical project protocol |
| Document status | Final 28 April 2015 including Amendment # 5 |

**Table of contents**

Protocol [**Fel! Bokmärket är inte definierat.**](#__RefHeading___Toc153632806)

Protocol Synopsis [**Fel! Bokmärket är inte definierat.**](#__RefHeading___Toc153632807)

1 Background [**Fel! Bokmärket är inte definierat.**](#__RefHeading___Toc153632808)

2 Purpose of project [**Fel! Bokmärket är inte definierat.**](#__RefHeading___Toc153632809)

3 Objectives [**Fel! Bokmärket är inte definierat.**](#__RefHeading___Toc153632810)

4 Project design [**Fel! Bokmärket är inte definierat.**](#__RefHeading___Toc153632811)

5 Population [**Fel! Bokmärket är inte definierat.**](#__RefHeading___Toc153632812)

6 Treatment [**Fel! Bokmärket är inte definierat.**](#__RefHeading___Toc153632813)

6.1 Immunosuppressive treatment [**Fel! Bokmärket är inte definierat.**](#__RefHeading___Toc153632814)

6.2 Identification number [**Fel! Bokmärket är inte definierat.**](#__RefHeading___Toc153632815)

6.3 Premature patient withdrawal [**Fel! Bokmärket är inte definierat.**](#__RefHeading___Toc153632817)

7 Visit schedule and assessments [**Fel! Bokmärket är inte definierat.**](#__RefHeading___Toc153632818)

7.1 Assessment schedule 10

7.2 Patient demographics/other baseline characteristics [**Fel! Bokmärket är inte definierat.**](#__RefHeading___Toc153632819)

7.3 Treatments [**Fel! Bokmärket är inte definierat.**](#__RefHeading___Toc153632820)

7.4 Efficacy [**Fel! Bokmärket är inte definierat.**](#__RefHeading___Toc153632821)

8 Data collection [**Fel! Bokmärket är inte definierat.**](#__RefHeading___Toc153632828)

9 Statistical analysis [**Fel! Bokmärket är inte definierat.**](#__RefHeading___Toc153632829)

10 References [**Fel! Bokmärket är inte definierat.**](#__RefHeading___Toc153632830)

11 Publication policy [**Fel! Bokmärket är inte definierat.**](#__RefHeading___Toc153632831)

# Protocol Synopsis

**Title of project:**

**MALTX – Projekt för uppföljning av MALignitet hos (TX)transplanterade patienter**

**Aim of project:**

The aim of the project is to improve the overall treatment and outcome of renal, pancreas or liver transplanted patients who have encountered a malignancy by a structured treatment programme for diagnosis and treatment of the malignancy, optimization of the immunosuppressive treatment, follow-up and evaluation of the programme.

**Objectives:**

The objective of the project is to improve the overall treatment and outcome of transplanted patients who have encountered a malignancy by a structured treatment programme for diagnosis and treatment of the malignancy, optimization of the immunosuppressive treatment, follow-up and evaluation of the programme.

**Population:**

The project population will consist of transplanted patients in Uppsala/Örebro region who have encountered a malignancy from September 2006 and prospectively up 2017 will be included. Based on previous experience we expect that 20-40 patients per year will be eligible to be included. All patients will be followed for at least 3 years.

**Inclusion/Exclusion criteria for the project:**

**Inclusion criteria**

- Patients willing and capable of giving written informed consent of participation in the project
- In Uppsala/Örebro region patients with a previous (2006 diagnosed or presently diagnosed or reoccurrence of a malignancy (other then basal cell carcinoma) will be included.

**Exclusion criteria**

- Patients with a primary liver cancer as cause of the transplantation

**Project design:**

All renal centres in the Uppsala region who are responsible for the follow-up of transplanted patients will receive a letter with full information about the project and a kind request to referrer all patients, with a diagnosed malignancy from September 2006 and onwards, to the Department of Transplantation at Uppsala University hospital.

Based on previous experience information regarding malignancies of the transplanted patients is missing in about 50% of the cases at the local renal centres and accordingly in the transplant registries.

A dedicated group of a responsible transplant surgeon/nephrologist, responsible specialist in oncology, responsible dermatologist and a project nurse will evaluate the patients from the Uppsala/Örebro region at the department of transplantation in Uppsala.

Once patient consent has been obtained baseline data will be collected. Further diagnostic measures to evaluate, stage and grade the tumour will be decided by the oncologist and a treatment plan will be drawn.

Based on the patient’s current immunosuppressant, renal function, grade of proteinuria, anti-cancer treatment needed or whether the patients will need surgery as part of the treatment for the tumour or not decision will be made to alter the immunosuppressant treatment.

The aim of altering the immunosuppressive treatment is to minimise the immunosuppressant in order not to further stimulate the growth and spread of the malignancy but also to secure enough immunosuppressant to prevent rejection and to maintain a good renal function.

A novel but acknowledged approach to this problem is to alter the immuno-suppressive treatment to PSI based immunosuppression. The calcineurin-inhibitors will be reduced to a minimum or be withdrawn and a Proliferation Signal Inhibitor (PSI) (everolimus or sirolimus) will be added. PSI treatment could have a direct effect on malignant cells and also exhibit an anti-angiogenesis effect by decreasing production of vascular endothelial growth factor (VEGF) while at the same time protecting the transplant from rejection.

Patients who at that time point are not suitable to have their immunosuppressive regimen changed due to interaction with anti-cancer treatment or scheduled surgery could at a later time point be offered to change their immunosuppressive treatment if the above no longer is true.

All patients will be evaluated regarding renal function, progression/regression/ reoccurrence of the malignancy and blood tests at baseline, 2-3 months after baseline and thereafter once yearly.

**Efficacy assessments in the project:**

- Patients and graft survival
- Regression/progression/reoccurrence of the diagnosed malignancy by physical exam, clinical investigations appropriate for respective malignancy (e.g. x-ray, scintigraphic methods, Magnetic Resonence (MR), Computer Tomography (CT), biomarkers (e.g PSA)
- De novo diagnosed malignancy
- Renal function as assesed by Serum-creatinine, u-protein, P-Cystatin-C, eGFR calculated by the Cockcroft-Gault formula
- Laboratory assessments

# 1 Background

The leading cause of death among allograft recipients has for a long time been cardiovascular complications. With the improved long-term outcome of kidney transplant recipient’s malignant tumours are becoming the leading cause of death. The risk of developing a malignant tumour increases with the increased amount of immunosuppressant, the duration of the immunosuppressive treatment and the age of the patient at the time of transplantation.

The mean age of the renal allograft recipients in Uppsala region are currently 58 years and the expected rate of finding a malignant tumour is one in every fifth patient, 5 years after transplantation **(Morath et al 2004).**

Most immunosuppressive agents used to prevent rejection in transplanted patients have been shown to increase the risk of malignancies. The aetiology is multifactorial and involves impaired immunosurveillance of neoplastic cells, direct oncogenic effect of the immunosuppressive drugs, reactivation of latent oncoviruses, chronic antigen stimulation of the graft and perhaps interference with DNA repair.

The risk of developing any type of malignant tumour is increased 3-5 times compared to the general population **(Morath et al 2004)**. For some specific types of malignancies such as cancer of anus and vulva, lymphoma, non-melanoma skin cancer and Kaposis sarcoma the increased risk compared to the normal population is 20-40 times higher. The malignant tumours of the transplanted patients also tend to progress faster and to metastasise earlier compared to malignancies in the general population. Malignant tumours of the transplanted population are also more difficult to treat due to the complex medical history of these patients and the multi drug treatment that most of these patients are treated with which can interfere with the planned oncological treatment.

The treatment of a malignancy in this patient’s population is a therapeutic dilemma also from the point of view that the immunosuppressive drugs are generally reduced or withdrawn in order to improve the immune system defence against the tumour cells which may result in deleterious effects on graft survival. In other words even if the patient survives the malignant disease the graft might not. Also good renal function is highly warranted to tolerate oncologic treatment.

Recent studies indicate that proliferation signal inhibitors (PSI) such as everolimus and sirolimus have both a direct effect on malignant cells and also exhibit an anti-angiogenesis effect by decreasing production of vascular endothelial growth factor(VGEF) while at the same time protecting the renal transplant from rejection (Koehl GE et al. 2004).

By reducing the calcineurin inhibitors to a minimum and adding a proliferation signal inhibitor the immunosuppressive treatment will both prevent rejection and keep a functioning transplant as well as having a neutral or maybe even a favourable effect on tumour regression.

This project aims to develop a structured treatment programme to try to improve the outcome in transplanted patients within the Uppsala/Örebro region who either have been treated for a malignancy within the last 12 months (September 2006), or have a reoccurrence of a previously known malignancy and those who prospectively encounter a malignancy during the time of the project.

# 2 Purpose of project

The treatment of a transplanted patient with a malignant disease is a medical challenge, which demands a multidisciplinary co-operation. The project aims to identify these patients and to structure the treatment and follow-up in order to improve the individual patient’s outcome. The aim of the project is to evaluate each transplanted patient with a malignant disease individually to optimize the immunosuppressive treatment in order to save the transplant as well as to optimize the oncological treatment to prolong the patients life. PSI treatment will be started in patients whose immunosuppressant according to the transplant surgeon and the oncologist safely can be altered in order to take advantage of the immunosuppressive and antitumour effects of these drugs.

# 3 Objectives

The objective of the project is to improve the overall treatment and outcome of transplanted patients who have encountered a malignancy by a structured treatment programme for diagnosis and treatment of the malignancy, optimization of the immunosuppressive treatment, follow-up and evaluation of the programme.

# 4 Project design

All renal centres in the Uppsala region who are responsible for the follow-up of transplanted patients will receive a letter with full information about the project and a kind request to referrer all patients with a diagnosed malignancy from September 2006 and onwards to the Department of Transplantation at Uppsala University hospital.

Based on previous experience in Uppsala region information regarding malignancies of the transplanted patients is missing in about 50% of the cases at the local renal centres and accordingly in the transplant registries. In order to identify the malignancies the local transplant registries in Uppsala will be crossrun with the Regional Oncologic Registry Uppsala Örebro.

A dedicated group of a responsible transplant surgeon/nephrologist, responsible specialist in oncology, responsible specialist in dermatology and a project nurse will evaluate the patients from Uppsala/Örebro region. Once patients consent has been obtained baseline data will be collected. Further diagnostic measures to evaluate, stage and grade the tumour will be decided by the oncologist and a treatment plan will be drawn.

Based on the patient’s current immunosuppression, renal function, grade of proteinuria, anti-cancer treatment needed or whether the patients will need surgery as part of the treatment for the tumour or not decision will be made to keep the immunosuppressant treatment unchanged or to alter it.

The aim of changing the immunosuppressive treatment is to minimise the immunosuppression in order not to further stimulate the growth and spread of the malignancy but also to secure enough immunosuppression to prevent rejection and to maintain a good renal function.

A thorough run-through of the patient’s different immunsuppressive drugs (Sandimmun Neoral/Prograf, Myfortic/Cellcept, Prednisolon, Azathioprin, Rapamune/Certican etc.) will be done in order to minimize the doses, alter or withdraw the drugs when possible.

Patients who at that time point are not suitable to have their immunosuppressive regimen changed due to interaction with anti-cancer treatment or scheduled major surgery could at a later time point be offered to change their immunosuppressive treatment if the above no longer is true.

All patients will be seen by the dedicated team for evaluation of renal function, progression/regression/reoccurrence of the malignancy and blood test at baseline. The info visit at 2 –3 months after baseline and annual visits for the next three years can when needed be replaced by information obtained from medical records in the patient´s local hospital. Evaluations with respect to the malignancy will be according to the standard procedures for each type of malignancy.

# 5 Population

The project population will consist of transplanted patients within the Uppsala/Örebro region who have encountered a malignancy from September 2006 and prospectively up until end of 2017. Based on previous experience we expect that 20-40 patients per year will be eligible to be included. All patients will be followed for at least 3 year.

**Inclusion/Exclusion criteria for the project:**

Inclusion criteria

- Patients willing and capable of giving written informed consent of participation in the project

In Uppsala Örebro region patients with a previous (during 2006) diagnosed or presently diagnosed or reoccurrence of a malignancy (other then basal cell carcinoma).

Exclusion criteria

Patients with a primary liver cancer as cause of the transplantation

# 6 Treatment

## Immunosuppressive treatment

In general the aim is to reduce all immunosuppressive treatment in all transplanted patients with a malignancy. The decision to change, reduce or withdraw any immunosuppressive treatment in these patient will be based on an individual overall evaluation of the patients situation, type of malignancy, time since transplantation and will be made by the responsible transplant surgeon/nephrologist in collaboration with the dedicated team for the project.

## 6.2 Identification number

Once a potential patient has been identified and the informed consent has been signed, the patient will be entered into the project. At that time, the patient will be assigned a unique identification number (enrolment number). Once assigned, patient identification numbers will not be reused and will remain with the patient throughout the project.

## 6.3 Premature patient withdrawal

Patients may voluntarily withdraw from the project or be dropped from it at the discretion of the investigator at any time.

Patients who are not able to appear for visits at the hospitals will not be withdrawn from the project. Contact will be taken with the patient’s ordinary hospital at home in order to complete the information and laboratory data needed for the follow-up within the project.

# Visit schedule and assessments

# 7:1 Assessment schedule

| **Visit** | | | **Visit 1** | | **Visit 2** | | **Visit 3**  ^± 1 month^ | | **Visit 4**  ^± 1 month^ | | **Visit 5**  ^± 1 month^ | |
| --- | --- | --- | --- | --- | --- | --- | --- | --- | --- | --- | --- | --- |
| **Day/week/year** | | | **Baseline** | | **Month 2-3** after baseline visit | | **Year 1** | | **Year 2** | | **Year 3** | |
| Informed consent **^1^** | | | X | |  | |  | |  | |  | |
| Vital signs **^2^** | | | X | | X | | X | | X | | X | |
| TX information | | | X | |  | |  | |  | |  | |
| Malignancy information **^3^** | | | X | | X | | X | | X | | X | |
| Immunosuppressive medication | | | X | | X | | X | | X | | X | |
| Hematology **^4^** | | | X | | X | | X | | X | | X | |
| Chemistry ^5^ | | | X | | X | | X | | X | | X | |
| Lipids ^6^ | | | X | | X | | X | | X | | X | |
| Fasting Glucose + C-peptide and amylase ^7^ | | | X | | X | | X | | X | | X | |
| CyA C0-h/Tac C0-h | | | X | | X | | X | | X | | X | |
| Eve/Sir C0-h | | |  | | X | | X | | X | | X | |
| Biomarkers **^8^** | | | X | | X | | X | | X | | X | |
| Renal function **^9^** | | | X | | X | | X | | X | | X | |
| Graft loss | | | As necessary | | | | | | | | | |
| Rejection | | | As necessary | | | | | | | | | |
|  | PEF 10 | X | | X | | X | | X | | X | |  |
|  | NTproBNP 11 | X | | X | | X | | X | | X | |  |
|  | UCG parameters, EF LVEDD Vksize 12 | X | | X | | X | | X | | X | |  |
|  | ISHT grading 13 | X | | X | | X | | X | | X | |  |
|  |  |  | |  | |  | |  | |  | |  |
|  |  |  | |  | |  | |  | |  | |  |

###### Legend:

1. The informed consent must be signed **prior to** the baseline assessments and the project entry.

2. Blood pressure, heart rate, weight, and height (only at baseline).

3. Baseline visit: Time of diagnosis, type of malignancy, localization, metastasis, previous and current treatment. Visit 2-5: Current status (i.e. cured, regression, status quo or progress) and current treatment.

1. Hemoglobin, WBC, platelets
2. ASAT, ALAT, ALP, Bilirubin, LDH, GT, SR, CRP, Sodium, Potassium, Calcium, Phosphate, Creatinine, Urea, Uric acid, HbA1c , Immuknow®

6. Total Cholesterol, HDL-C, LDL-C, Triglycerides

7. Only in pancreas or islet transplanted patients

1. CEA Ca19-9 PSA and other relevant markers of malignancy according to the oncologist and the local treatment guidelines.
2. eGFR (Cystatin-C), U-albumin (quantitative 24h) and/or albumin/creatinine ratio (morning sample)
3. PEF (Peak Expiratory Flow) only in lung transplanted patients.
4. Only in heart transplanted patients.
5. UCG parameters EF and LVEDD (and VK size) only in heart transplanted patients according to the local treatment guidelines.
6. ISHT grading (0-3 and/or AMR yes/no) if a biopsi has been performed according to the local treatment guidelines. Only in heart transplanted patients.

## Patient demographics/other baseline characteristics

After the informed consent document has been signed and it has been determined that the patient is eligible to participate in the project, baseline assessment information will be obtained and entered into eCRF. This information will include demographics (date of birth and gender) and relevant medical history/current medical conditions.

Information about the transplantation will be obtained: Date of transplantation, first, second or third transplantation, kind of donation (living or deceased), disease leading to transplantation.

Information about the malignancy will be obtained: Time of diagnosis, type of malignancy, localization, metastasis, and previous and current treatment.

## Treatments

Initial dosages of all immunosuppressive drugs will be recorded in the eCRF at baseline. Current immunosuppressive drugs name of drug, doses, and reason for treatment will be recorded at each follow-up visit within the project. The stop date and reason for stopping any immunosuppressive drugs will be recorded irrespectively of if it occurs at a project visit or in-between project visits.

All types of treatment for the malignancy will be recorded in the eCRF, medical/radiation/surgical, name of drugs used, start of drug treatment, status of treatment (ongoing, completed, prematurely stopped and reason for stopping).

## 7.4 Efficacy

Patients and graft survival

Patient survival will be calculated as number of days from first malignancy diagnosed.

Graft survival will be calculated both as number of days between TX and graft loss and time of detection of malignancy and graft loss (i.e. return to dialysis) or death.

Regression/Progression/Reoccurrence

Regression/progression/reoccurrence of the diagnosed malignancy by clinical investigation appropriate for respective malignancy (e.g. x-ray, scintigrafic methods, Magnetic Resonase (MR), Computer Tomography (CT), biomarkers (e.g. PSA)

Renal function

Deterioration/improvement in renal function will be measured as changes in S-creatinine, P-Cystatin-C and U-albumin from baseline, during the follow-up and until end of 3-year follow-up for all patients in the project. Renal function will also be calculated by use of the Cockcroft-Gault formula:

Calculated value of GFR in ml/min - Cockcroft-Gault formula

For men: GFR**=**$\frac{(140-Age)\times Body Weight \left[kg\right]}{1.23\times Serum Creatinine \left[umol/l\right]}$

For women: GFR**=**$\frac{0,85\times(140-Age)\times Body Weight \left[kg\right]}{1.23\times Serum Creatinine \left[umol/l\right]}$

Vital signs

Vital signs variables include measurements of systolic and diastolic blood pressures, heart rate, height (only at baseline) and body weight (at every visit).

Laboratory evaluations

Hematology

Hemoglobin, WBC, and platelet count.

Blood chemistry

- ASAT, ALAT, ALP, Bilirubin, LDH, TC, HDL-C, LDL-C, TG, SR, CRP, Sodium, Potassium, Calcium, Phosphate, Creatinine,Urea, Uric acid, Cystatin-C, HbA1c, Immuknow®
  - If the total bilirubin concentration is greater than 3 times the upper limit of normal, total bilirubin should be differentiated into direct reacting bilirubin and none-direct reacting bilirubin

Fasting C-peptide + glucose and amylase (only in patients with pancreas

or islet graft

NTproBNP

**Urine test**

U-albumin, quantitative (24 h) and/or albumin/creatinine ratio (morning sample).

**Biomarkers**

Biomarkers taken in routine clinical practice will be registered in the project.

**Blood concentration of immunosuppressants**

C0 blood concentration of immunosuppressants taken in routine practice will be registered in the project**.**

**Other tests**

PEF (Peak Expiratory Flow)

UCG parameters EF and LVEDD (and VK size)

ISHT grading (0-3 and/or AMR yes/no)

**8 Data collection**

Data from the project will be captured in an eCRF. The database and data capture will be managed by the company Pharma Consulting Group AB (PCG).

Pharma Consulting Group AB

Uppsala Science Park

Dag Hammarskjölds väg 40C

SE-751 83 UPPSALA

Sweden

The responsible transplant surgeon/nephrologist or project nurse will enter data on the eCRFs.

The database will be programmed to give password-protected access to the various contributors to the project:

# 9 Statistical analysis

All data will be summarized using frequency distribution (for categorical variables) and descriptive statistics of mean, median, maximum, minimum, and standard deviation (for continuous variables).

Survival and tumour free survival will be calculated for each individual tumour form and compared with corresponding data for these tumours of the general population. Data will be obtained from the Swedish national cancer registry and ROC (regionalt onkologiskt centrum in Uppsala).

The ITT population will consist of all patients who have been to the baseline visit evaluation and who have at least baseline laboratory data.

# 10 References

Koehl GE, Andrassy J, Guba M, et al. Rapamycin protects allograft from rejection while simultaneously attacing tumors in immunosuppressive mice. Transplantation 2004;77:1319

**Christian Morath, Martina Mueller, Hartmut Goldschmidt** et al. Malignancy in Renal Transplantation. J Am Soc Nephrol 2004;15:1582-8

11 Publication policy

The steering committee will review, evaluate and write a publication of the data from the project.
